# Activation of a Habenulo–Raphe Circuit Is Critical for the Behavioral and Neurochemical Consequences of Uncontrollable Stress in the Male Rat

**DOI:** 10.1523/ENEURO.0229-16.2016

**Published:** 2016-10-17

**Authors:** Samuel D. Dolzani, Michael V. Baratta, Jose Amat, Kara L. Agster, Michael P. Saddoris, Linda R. Watkins, Steven F. Maier

**Affiliations:** 1 Department of Psychology and Neuroscience and the Center for Neuroscience, University of Colorado Boulder, Boulder, Colorado 80309; 2 Institute for Behavioral Genetics, University of Colorado Boulder, Boulder, Colorado 80309

**Keywords:** amygdala, habenula, optogenetics, raphe, serotonin, stress

## Abstract

Exposure to uncontrollable stress [inescapable tailshock (IS)] produces behavioral changes that do not occur if the stressor is controllable [escapable tailshock (ES)] an outcome that is mediated by greater IS-induced dorsal raphe nucleus (DRN) serotonin [5-hydroxytryptamine (5-HT)] activation. It has been proposed that this differential activation occurs because the presence of control leads to top–down inhibition of the DRN from medial prefrontal cortex (mPFC), not because uncontrollability produces greater excitatory input. Although mPFC inhibitory regulation over DRN 5-HT activation has received considerable attention, the relevant excitatory inputs that drive DRN 5-HT during stress have not. The lateral habenula (LHb) provides a major excitatory input to the DRN, but very little is known about the role of the LHb in regulating DRN-dependent behaviors. Here, optogenetic silencing of the LHb during IS blocked the typical anxiety-like behaviors produced by IS in male rats. Moreover, LHb silencing blocked the increase in extracellular basolateral amygdala 5-HT during IS and, surprisingly, during behavioral testing the following day. We also provide evidence that LHb–DRN pathway activation is not sensitive to the dimension of behavioral control. Overall, these experiments highlight a critical role for LHb in driving DRN activation and 5-HT release into downstream circuits that mediate anxiety-like behavioral outcomes of IS and further support the idea that behavioral control does not modulate excitatory inputs to the DRN.

## Significance Statement

Uncontrollable stressors produce dorsal raphe nucleus (DRN)-dependent behavioral changes that are prevented if subjects are provided with a controlling response over the stressor. It is proposed that controllable stress is protective because it leads to direct medial prefrontal cortex inhibition of DRN serotonin (5-hydroxytryptamine) neurons rather than reducing excitatory inputs to the DRN. However, the critical excitatory inputs to DRN that mediate the behavioral effects of uncontrollable stress have received little study. Here we show that lateral habenula (LHb) activation is necessary for mediating the DRN and the behavioral outcomes produced by uncontrollable stress. Moreover, excitatory LHb input during the stressor was not reduced by behavioral control.

## Introduction

Exposure to adverse events impacts the functioning of an organism at the behavioral and neurochemical levels (Christianson et al., 2009). However, individuals may react quite differently to seemingly similar adverse events (Alleva and Francia, 2009; Maier 2015). The degree of behavioral control over the event potently influences the outcome of the exposure (Maier and Watkins, 2005; Drugan et al., 2013; Hartley et al., 2014). Rats exposed to inescapable (uncontrollable) tailshock (IS), but not equivalent escapable (controllable) tailshock (ES), show increased anxiety (Maier, 1990; Minor et al., 1994; Baratta et al., 2007; Christianson et al., 2009) and depression-like (Weiss and Simson, 1986; Maier et al., 1995) behavioral changes.

The behavioral changes produced by uncontrollable stressors, such as IS, are mediated, at least in part, by serotonergic [5-hydroxytryptamine (5-HT)] neurons located in the mid to caudal dorsal raphe nucleus (DRN). IS and other uncontrollable stressors activate and sensitize these neurons, as indicated by the increased Fos expression in 5-HT-labeled cells (Grahn et al., 1999; Takase et al., 2004), and the release of 5-HT within the DRN (Maswood et al., 1998) and projection regions, such as the basolateral amygdala (BLA), a proximal mediator of IS-induced, anxiety-like behavior (Amat et al. 1998; Christianson et al., 2010). Pharmacological inhibition of the DRN during IS prevents the behavioral sequelae of IS (Will et al., 2004), while pharmacological activation in the absence of IS produces them (Maier et al., 1995). The intense activation of DRN 5-HT neurons by IS leads to their sensitization (Rozeske et al., 2011), so that later exposure to nonaggressive juvenile social investigation (JSI) leads to the release of exaggerated amounts of 5-HT in projection regions that are the proximate mediators of the behaviors, thereby producing the behavioral changes.

As required by the idea that DRN 5-HT activation is critical in mediating the consequences of IS, exactly equal amounts of ES do not produce DRN activation or behavioral changes typically associated with DRN activation (Maswood et al., 1998). Differential activation of the DRN by IS relative to ES could be caused by (1) excitatory input to the DRN produced by IS, but not ES; or (2) excitatory input during both, but also inhibitory input during ES. A variety of evidence has supported the second of these possibilities and has documented an inhibitory input during ES (for review, see Maier, 2015). This work has led to a proposed model (Maier, 2015) in which ES and IS provide equal excitatory inputs to the DRN, whereas ES provides a selective inhibitory input from the prelimbic region of the ventral medial prefrontal cortex. However, the excitatory inputs that drive DRN 5-HT activation have not been extensively explored.

A strong glutamatergic projection from the lateral habenula (LHb) to DRN has been noted in many studies (Aghajanian and Wang, 1977; Kalén et al., 1985; Araki et al., 1988; Sego et al., 2014). This is of significant interest because many recent studies have converged on the LHb as a key structure involved in aversion, mood disorders, post-traumatic stress disorder, stress, and psychosis (Matsumoto and Hikosaka, 2007; Hikosaka, 2010; Lammel et al., 2012; Gill et al., 2013; Li et al., 2013; Meye et al., 2013; Nair et al., 2013). Interestingly, Amat et al. (2001) reported that electrolytic lesions of the habenular complex prevented the typical DRN 5-HT increase during IS and the later behavioral changes produced by IS (Amat et al., 2001) .

However, the lesions in the study by Amat et al. (2001) encompassed both the LHb and medial habenula (MHb), and these two regions of the habenula have different inputs, outputs, and functions (Sutherland, 1982; Aizawa et al., 2012; Zhao et al., 2015). Additionally, electrolytic lesions would have destroyed fibers of passage, and the habenula was inactive during both IS and later behavioral testing. Finally, Amat et al. (2001) did not examine habenula activation by IS and ES.

Here we explore whether optogenetic silencing of the LHb only during the tailshock intervals of IS prevents the increases in BLA extracellular 5-HT during IS, as well as increased BLA 5-HT and anxiety-like behavior during JSI 24 h later. We also examine whether ES and IS do, or do not produce equivalent activation of DRN-projecting LHb neurons.

## Materials and Methods

### Rats

Adult male Sprague Dawley rats (250–300 g; Harlan) were singly housed on a 12 h light/dark cycle (lights on at 07:00 A.M. and off at 07:00 P.M.). Rats were housed with free access to food and water, and were allowed to acclimate to colony conditions for 7 d prior to surgical or experimental procedures. All experiments were performed between 9:00 A.M. and 5:00 P.M. All animal procedures were approved by the Institutional Animal Care and Use Committee at the University of Colorado, Boulder, and conformed to National Institutes of Health *Guidelines on the Care and Use of Laboratory Animals*.

### Virus

Adeno-associated virus (AAV) vector was used to target third-generation halorhodopsin (NpHR)-enhanced yellow fluorescent protein (eYFP) or control (eYFP) expression to LHb pyramidal neurons. CaMKIIα::eNpHR-eYFP and CaMKIIα::eYFP cassettes were packaged in AAV vectors serotyped with AAV5 coat proteins (titers, 3.0–6.0 × 10^12^ genome copies/ml) by the Vector Core at the University of North Carolina at Chapel Hill.

### Surgical procedure

**Figure 1.**
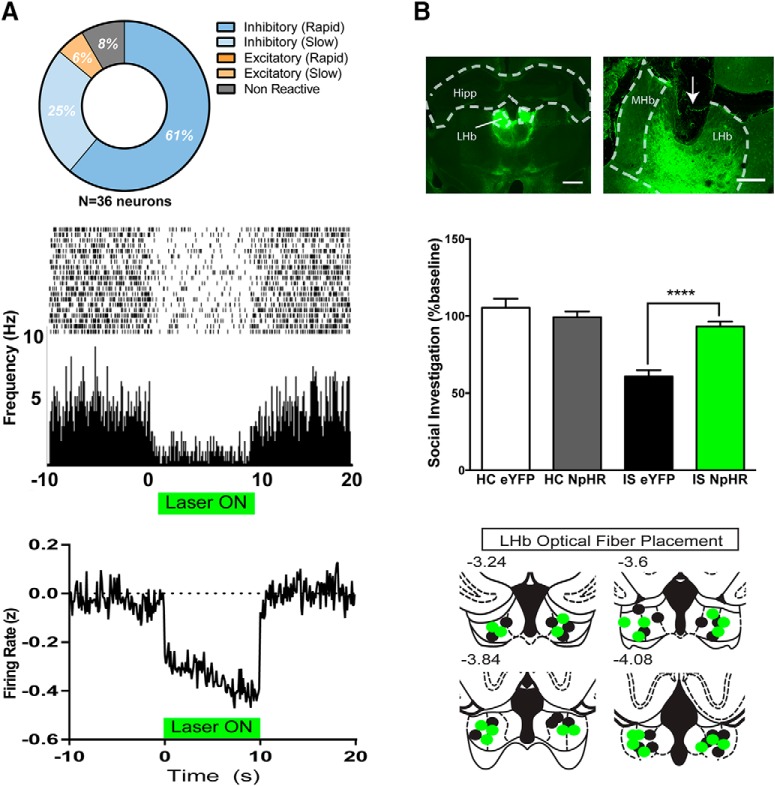
Optical inhibition of the LHb during IS prevents later anxiety-like behavior. ***A***, Top left, Changes in LHb firing rate in response to continuous (10 s) green light illumination of LHb in rats expressing NpHR. Middle, Raster plot and perievent histogram showing the firing rate of a representative LHb neuron in a rat expressing NpHR in the LHb (bin size, 100 ms). Green bar represents continuous laser illumination. Bottom, The *z*-normalized firing rate of all valid neurons recorded during green light illumination of LHb (*n* = 36), ***B***, Coronal micrographs demonstrating NpHR expression in the LHb in a rat that received IS plus optical silencing (IS+NpHR). Top left, Bilateral NpHR 3.0-eYFP expression (4× mosaic; scale bar, 1000 μm) and (top right) unilateral NpHR3.0-eYFP expression (inverted arrow indicates tissue damage from optical fiber implant (10×; scale bar, 200 μm). Middle, JSI data expressed as the percentage of baseline for rats previously injected with either eYFP or NpHR and later exposed to IS or HC treatment with green light illumination of the LHb (*n* = 10-11/group). Bottom, Diagram indicating the location of optical fiber tips in the LHb of rats that received IS plus NpHR 3.0 silencing (green dots) or IS plus eYFP (black dots). Position relative to bregma is denoted above individual images. Hipp, Hippocampus.

**Figure 2.**
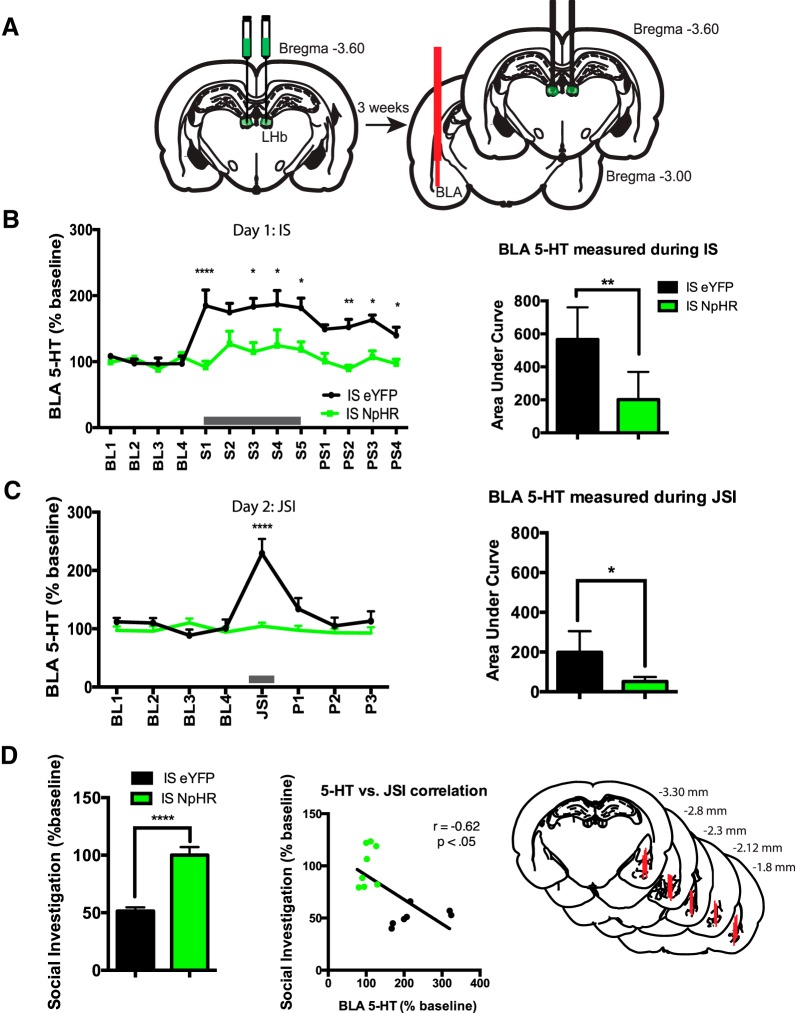
Optical inhibition of the LHb during IS prevents the neurochemical consequences of the stressor. ***A***, Rats were injected with NpHR or eYFP, and implanted with LHb optical fibers and BLA microdialysis probes 3 weeks later. ***B***, Left, BLA 5-HT levels measured during IS in rats that received IS plus NpHR or IS plus eYFP with green light delivery during tailshocks. Right, Area under the curve for BLA 5-HT levels measured during IS (arbitrary units, relative to average of baseline BLA 5-HT samples). ***C***, Left, BLA 5-HT measured during JSI in rats that previously received IS plus NpHR or IS plus eYFP with green light delivery during tailshocks. Right, Area under the curve for BLA 5-HT measured during JSI (arbitrary units, relative to average of baseline BLA 5-HT samples). ***D***, Left, JSI data for all rats that received IS plus NpHR or IS plus eYFP (*n* = 7-8/group). Middle, Correlation between BLA 5-HT levels measured during JSI and social investigation. Right, Schematic of BLA microdialysis probes for all rats included in statistical analysis. Position relative to bregma is denoted.

For optogenetic experiments, rats were anesthetized under isoflurane anesthesia (2%). Two small windows (1 × 1 mm) were drilled into the skull, and NpHR or control eYFP was bilaterally microinjected into the LHb [anteroposterior (AP), −3.6 mm relative to bregma; dorsoventral (DV), −5.0 mm from skull surface; mediolateral (ML), ±0.5 mm relative to midline] using a 10 μl Hamilton syringe and a 31 gauge metal needle with a 45° beveled tip. The total injection volume (0.5 μl) and flow rate (0.1 μl/min) were controlled with a microinjection pump (UMP3-1, World Precision Instruments). This injection volume was chosen based on pilot experiments in which robust eYFP expression was observed within the LHb 3 weeks after a 0.5 μl injection, while maintaining minimal infection of surrounding brain regions (data not shown). Following injection, the needle was left in place for an additional 10 min to allow for virus diffusion, after which the needle was withdrawn. The small scalp incision was closed using Vetbond (3M). Preoperative antibiotic (0.25 ml/kg, s.c.; Combi-Pen-48) and a postoperative analgesic (2 mg/kg, s.c.; Meloxicam) was administered to all rats. One week prior to the time of behavioral experimentation (3 weeks after viral injection), subjects received a second surgical procedure in which a custom-made light-delivery fiber optic (200 μm diameter core; 0.39 numerical aperture; Thorlabs) was chronically implanted bilaterally into the LHb. For rats that received microdialysis, a single unilateral microdialysis guide (CMA) was implanted into the BLA (AP, −3.0; ML, 4.8; DV, −6.2) during the fiber optic implantation surgery, using parameters identical to those previously described (Christianson et al., 2013). A plastic stylet remained in the guide until microdialysis probes were inserted at the time of experimentation. The optical fiber implants and microdialysis probes were adhered to the skull using anchor screws and acrylic cement. NpHR or eYFP expression, optical fiber placement, and/or microdialysis probe placement were determined after the completion of behavioral testing (Figs. 1*B*, 2*D* ; Paxinos and Watson, 2007). Only rats with accurate virus expression, bilateral optical fiber placement, and accurate microdialysis probe placement were used for statistical analysis (Tables 1, 2, 3).

**Table 1.** Statistical analyses performed in all experiments

Figure	Data structure	Type of test/comparisons	*p* Value
1A		See below	
1B	Normal distribution	Two-way ANOVA (stress treatment × virus type)	Main effect of virus type: *p* = 0.0044
		Main effect of stress treatment: *p* < 0.0001	
		Sidak’s multiple-comparisons test	Interaction: *p* < 0.0001
		Home cage–inescapable Stress	95% CI:
		eYFP	30.24–58.97
		NpHR	−7.976 to 20.10
2B	Normal distribution	Two-way repeated-measures ANOVA	Main effect virus type: *p* = 0.0007
		Sidak’s multiple comparisons test	Main effect time: *p* < 0.0001
		eYFP – NpHR	Interaction: *p* = 0.0002
		BL1	95% CI:
		BL2	−43.11 to 65.40
		BL3	−59.67 to 48.84
		BL4	−47.73 to 60.78
		S1	−66.85 to 41.66
		S2	33.82–142.3
		S3	−9.440 to 99.07
		S4	7.267–115.8
		S5	1.613–110.1
		PS1	0.8602–109.4
		PS2	−5.678 to 102.8
		PS3	13.43–121.9
		PS4	2.628–111.1
		Independent samples *t* test (AUC)	0.5271–109.0
		T, df	*p* = 0.0023
			*t* = 3.859 df = 12.00
2C	Normal distribution	Independent-samples *t* test for baseline JSI 5-HT values	Maine effect virus type: *p* = 0.0029
		Two-way repeated-measures ANOVA	Main effect time: *p* < 0.0001
		Sidak’s multiple-comparisons test	Interaction: *p* < 0.0001
		eYFP – NpHR	95% CI:
		BL1	−31.60 to 60.99
		BL2	−32.35 to 60.23
		BL3	−67.83 to 24.76
		BL4	−41.90 to 50.69
		JSI	78.49–171.1
		PI1	−9.353 to 83.23
		PI2	−34.55 to 58.03
		PI3	−25.61 to 66.98
		Independent-samples *t* test (AUC)	*p* = 0.0102
		T, df	*t* = 3.580 df = 6.479
2D	Normal distribution	Independent-samples *t* test	*p* < 0.0001
		T, df	*t* = 6.344 df = 9.716
2D	Normal distribution	Linear regression	*p* = 0.0125
			*R*^2^ = 0.3921
			*R* = −0.6257
		T, df	*t* = 6.344 df = 9.716
3	Normal distribution	Ordinary one-way ANOVA	*p* = 0.0065
		Tukey’s *post hoc* test	95% CI
		MHb Fos 0 h	0.03556 to 2.589
		HC vs ES	0.4731 to 3.027
		HC vs IS	-0.8394 to 1.714
		ES vs IS	*p* < 0.0001
		LHbM Fos 0 h	−37.59 to −19.54
		HC vs ES	−34.15 to −16.10
		HC vs IS	−5.587 to 12.46
		ES vs IS	*p* = 0.1416
		LHbL Fos 0 h	*p* = 0.3300
		MHb Fos 2 h	*p* = 0.0013
		LHbM Fos 2 h	95% CI:
		HC vs ES	−38.21 to −8.789
		HC vs IS	−34.40 to −4.977
		ES vs IS	−10.90 to 18.52
		LHbL Fos 2 h	*p* = 0.1888
		MHb Fos 4 h	*p* = 0.8780
		LHbM Fos 4 h	*p* = 0.8652
		LHbL Fos 4 h	*p* = 0.0348
		HC vs ES	95% CI:
		HC vs IS	−3.706 to 0.4562
		ES vs IS	−4.331 to −0.1688
			−2.706 to 1.456
4	Normal distribution	Ordinary one-way ANOVA	95% CI:
		Tukey’s *post hoc* test	*p* = 0.6651
		Total FG MHb	*p* = 0.7163
		Total FG LHbM	*p* = 0.5220
		Total FG LHbL	*p* = 0.9074
		Total Fos-IR MHb	*p* < 0.0001
		Total Fos-IR LHbM	95% CI:
		HC vs ES	−16.94 to −6.726
		HC vs IS	−18.73 to −8.517
		ES vs IS	−6.899 to 3.316
		Total Fos-IR LHbL	*p* = 0.0109
		HC vs ES	95% CI:
		HC vs IS	−8.395 to −1.022
		ES vs IS	−7.020 to 0.3534
		Total % double-label MHb	−2.312 to 5.062
		Total % double-label LHbM	*p* = 0.4152
		HC vs ES	*p* < 0.0001
		HC vs IS	95% CI:
		ES vs IS	−21.49 to −7.661
		Total % double-label LHbL	−18.44 to −4.614
			−3.866 to 9.960
			*p* = 0.0764

**Table 2.** Electrophysiology statistics for all recorded neurons

Cell firing rate (Hz)	ANOVA	BL vs laser	BL vs post	Laser vs post
31.05	0.68961273	n.s.	n.s.	n.s.
2.01	0.100469015	0.891754475	0.103118866	0.239922898
1.11	7.62002E-10	0.57315978	0.000124021	0.000124007
4.23	4.98676E-05	0.000171631	0.017046495	0.023234465
22.98	7.71135E-08	0.000149437	0.183615744	0.000150392
19.445	1.55431E-15	0.000124006	0.000187433	0.000124006
18.46	3.07286E-09	0.000124007	0.014428278	0.000139218
19.12	8.43769E-15	0.000124006	0.000355866	0.000124006
1.63	4.95913E-11	0.000124014	0.106283736	0.000124006
6.035	0	0.000124006	0.3640062	0.000124006
8.495	0	0.000124006	0.000124006	0.000124006
7.31	2.77556E-14	0	0.000124007	0
2.315	9.95919E-08	0.000759905	0.012110937	0.000124017
2.005	1.39015E-05	0.000133681	0.298443736	0.001375559
1.975	0.000155928	0.000359192	0.768212169	0.002040156
6.205	0	0.000124331	0.586462726	0.308035137
1.405	3.94043E-09	0.000791913	0.000630968	0.000124007
3.655	0	0.000124006	0.217018108	0.000124006
8.675	0	0.000124006	0.315496719	0.000124006
4.735	1.11022E-16	0.000124006	0.000202196	0.000124006
2.057894737	0	0.000126757	0.722558918	0.000126757
4.163157895	1.0224E-08	0.00014295	0.034134356	0.000126757
9.147368421	2.22045E-16	0.000126757	0.00012686	0.000126757
2.19	1.82944E-08	0.000124183	0.897361366	0.000124035
3.585	2.02568E-10	0.000124183	0.897361366	0.000135351
2.34	0	0.000124006	0.000124006	0.000124006
2.43	7.2915E-12	0.000124007	0.000124006	0.107882478
16.19	1.08128E-05	0.000155138	0.200273849	0.000538695
8.645	6.02274E-12	0.000124006	0.004329425	0.000124034
24.94	7.95227E-08	0.00012406	0.512140855	0.000127955
20.895	1.20682E-10	0.000124006	0.000322419	0.000161763
1.23	0.000437388	0.001932198	0.990424063	0.001355133
2.78	5.77942E-06	0.00176329	0.135744677	0.00012667
7.11	0	0.000124006	0.68520793	0.000124006
1.185	2.26193E-10	0.167593762	0.000124035	0.000124006
2.342105263	7.6863E-10	0.000126957	0.104059273	0.000126757

**Table 3.** The *z*-normalized firing rate for all recorded neurons

BL	Laser	Recovery
−9.21485E-17	−0.068442491	−0.034221245
−9.10383E-17	0.019584164	0.090087152
−1.04812E-16	−0.038779231	0.268952733
−9.99201E-18	−0.455220728	−0.233230373
−5.96745E-17	−1.024094554	−0.207156056
−0.132067429	−0.919189306	−0.410609643
−0.102381307	−0.789798657	−0.357359516
−0.100727222	−0.850484148	−0.342746023
−0.015070323	−0.262474797	0.04521097
−0.047670764	−0.632476897	−0.09332727
−0.034264811	0.027295697	0.554625337
−0.077528279	−0.439972986	−0.200927458
−0.009444506	−0.11963041	0.073457269
−0.019688117	−0.189932426	−0.068329348
−0.015790119	−0.205271549	−0.078950596
−3.94129E-17	−0.615002166	0.245870431
1.02141E-16	−0.190553967	0.194579755
3.33067E-17	−0.544864081	0.05771146
−6.55032E-17	−0.690738882	−0.054442473
−1.73195E-16	−0.395196601	0.187793805
−0.017634339	−0.285847671	−0.003923753
−0.032238634	−0.232802881	0.067656299
−0.051458356	−0.367047892	0.193812657
1.11022E−17	−0.201257541	0.013916745
−7.54952E-17	−0.144895939	0.198278654
−0.031527833	0.63160759	0.265884726
4.55191E-17	0.341549335	0.432560984
−1.17684E-16	−0.588112809	−0.160488531
−0.073112074	−0.45375906	−0.18858273
−0.067924124	−0.424629951	−0.145849345
−0.094099006	−0.442713419	−0.259444403
−0.021839826	−0.123759015	−0.004367965
1.29341E-16	−0.137644887	0.072012888
8.46545E-17	−0.391554728	0.022322843
−1.9984E-17	−0.052093519	0.193324838
−0.024702377	−0.245136395	0.047017782

### *In vivo* electrophysiological recording

Single-unit recordings were performed in a small subset of animals (*n* = 2) under urethane anesthesia (1.5 g/kg, i.p.) 3 weeks after viral injection. Simultaneous optogenetic silencing and electrophysiological recording of LHb pyramidal neurons transduced with either NpHR or eYFP were performed using a custom optrode consisting of a single tungsten electrode affixed to an optical fiber. The fiber tip was positioned 500 μm dorsal to the recording tip so that light would be emitted in a cone that illuminated neurons in close proximity to the recording tip. The optical fiber was coupled to a green diode laser (λ = 532 nm; Shanghai Laser & Optics Century) and yielded a fiber tip intensity of ∼10 mW/mm^2^, as determined by an optical power meter (Newport) prior to insertion into brain. A small craniotomy (4 × 4 mm) was made over the viral injection site. The optrode was driven through the LHb in 0.1 mm increments using a stereotaxic device (Kopf), starting from the dorsal boundary of the LHb at a depth of ∼4.0 mm from the brain surface (∼4.5 mm from skull surface). Detailed methods for electrophysiological recordings have been described previously (Saddoris et al., 2011; Saddoris and Carelli, 2014). Briefly, neural signals were amplified via a unity gain headstage, digitally sampled at 24 kHz, and recorded using the OmniPlex platform (Plexon). The resultant unit activity was isolated (Offline Sorter, Plexon), and aligned to time-locked laser onset and offset events (NeuroExplorer, NEX Technologies). The timing of laser presentations was controlled via computer (Med Associates). The schedule of light delivery followed a 10 s baseline period (no light delivery), and 10 s continuous light delivery and 10 s post-light delivery periods. Twenty consecutive trials were performed at each recording depth. Graphing was performed using Neuro Explorer software or Prism (GraphPad), and data analysis was performed using Prism (GraphPad).

Data from these recordings were assessed first for light reactivity using a one-way repeated-measures ANOVA for the average firing rate for each neuron over three consecutive 10 s epochs (i.e., baseline, light, and post-light recovery). For cells that showed light-related deflections in activity (i.e., Tukey’s *post hoc* tests on main effects of light delivery indicated a significant difference between baseline and the light delivery period), we then assessed the latency at which the light first induced changes in neural activity. Here neural activity during light delivery was separated into consecutive 100-ms-wide events (bin size, 100 ms) and was compared to the average baseline using paired *t* tests. The first bin that was significantly different from baseline and followed by a second significantly different bin was determined to be the latency of light-induced changes in activity. Previous work has shown that NpHR-expressing neurons display extremely rapid (<2 s) decreases in neural activity in the presence of light. Thus, changes in activity that showed latencies of changed neural activity that was >2 s were presumed to be due to nonspecific network effects (i.e., light was acting on cells connected to, but distinct from, the recorded unit) and were counted separately for analysis of firing kinetics. To assess population level activity, we normalized firing rates to *z*-scores using the mean and SD from the baseline period in each neuron, and then averaged the resulting normalized rates. To ensure the ability to statistically detect inhibitions, we only used cells with a basal firing rate of at least 1 Hz for all of the analyses described above. The range of average latencies and firing rates is expressed as the mean ± SEM.

### Experiment 1: effect of optogenetic silencing of LHb during inescapable tailshock on later juvenile social investigation

#### Inescapable tailshock procedure

Rats previously injected with NpHR or eYFP received 100 5 s inescapable tailshocks in Plexiglas boxes (14 × 11 × 17cm), as described by Christianson et al. (2013). The tail of the rat was secured to a Plexiglas post protruding from the rear portion of the box using medical tape, and copper electrodes were placed around the tail. Shock was delivered to the tail of the rat with increasing intensity as the shock session progressed (33 trials at 1.0 mA, 33 trials at 1.3 mA, and 34 trials at 1.6 mA). Shock was delivered with an average intertrial interval (ITI) of 60 s. Rats were removed from the Plexiglas boxes and placed in their home cage immediately after the last tailshock.

#### Green laser light delivery

For NpHR-mediated silencing of LHb activity or eYFP light delivery (control), the implanted optical fibers were connected to a patch cable that interfaced with an fiber optic connector/physical contact (FC/PC) fiber optic rotary joint (Doric Lenses), which interfaced with a 532 nm solid-state green laser (Shanghai Laser and Optic Co.) outside the wheel turn box. Rats were restrained in Plexiglas boxes, as described above. One hundred 5 s inescapable tailshocks with an average ITI of 60 s were delivered over the duration of the shock session (approximately 1 h and 45 min). Continuous green light delivery was achieved for the duration of each 5 s tailshock by routing the electrical signal generated by the animal shocker through a custom program designed using LabView (National Instruments) that triggered the laser for 5 s upon receiving the tailshock signal. Separate groups of home-cage rats injected with either NpHR or eYFP received the same duration and temporal pattern of light delivery to the LHb while in their home cage.

#### Juvenile social investigation

JSI testing was conducted as previously described (Christianson et al., 2009). In a behavioral testing room, each experimental subject was singly assigned to an empty plastic cage with shaved wood bedding and a wire lid. Experimental subjects remained in the test cage for 1 h prior to introducing a juvenile (28 ± 2 d old) male conspecific. An observer, blind to treatment, recorded exploratory behavior (allogrooming, sniffing, and pinning) initiated by the experimental subject during a 3 min baseline test, which occurred 24 h prior to IS or home-cage control (HC) treatment. Twenty-four hours after the last tailshock or HC, experimental subjects were tested again using parameters identical to those used for the baseline test. Juveniles were used for multiple tests, but never with the same rat twice. JSI test scores were reported as a percentage of baseline social investigation time on the test day.

### Experiment 2: effect of optogenetic silencing of LHb during inescapable tailshock on BLA 5-HT release during IS and subsequent juvenile social investigation

#### Inescapable stress procedure with simultaneous *in vivo* microdialyisis

Twenty-four hours before IS, rats previously injected with NpHR or eYFP were taken to the microdialysis room where they were placed in a Plexiglas microdialysis bowl (Bioanalytical Systems). Microdialysis probes (CMA 12; molecular weight cutoff, 20 kDa; 2 mm) were inserted into the guide, and Ringer’s solution (145 mmol/L NaCl, 2.7 mmol/L KCl, 1.2 mmol/L CaCl) was perfused through the probes at a flow rate of 2 μl/min. At the same time, the implanted optical fibers were connected to a patch cable that interfaced with an FC/PC fiber optic rotary joint (Doric Lenses), which interfaced with a 532 nm solid-state green laser. Microdialysis tubing and optical fibers were protected by a metal spring coil that surrounded the microdialysis tubing and fibers, which was routed into an inverted conical cap mounted on the head of the rat. After 18 h of acclimation, the microdialyisis pump flow rate was increased to 1.5 μl/min. A JSI baseline measurement was made 3 h prior to the start of microdialysis sample collection. During this baseline test, a juvenile was placed in the microdialysis bowl for a total of 5 min, and social interaction (licking sniffing, grooming) was recorded by an observer who was blind to condition (Christianson et al., 2010). Following a 2 h equilibration period after the JSI baseline measure, 13 dialysate samples were collected at 20 min intervals. Four prestress baseline samples were collected while rats were freely moving in microdialysis bowls. During the fifth sample, rats were placed in Plexiglas boxes that accommodated the microdialysis tubing and optical fibers for the duration of the IS procedure. All IS rats received 100 tailshocks (see the IS procedure described above). Continuous green light (8–10 mW intensity at fiber tip) delivery was time locked to each tailshock, such that light was delivered to the LHb only during tailshock. A total of five dialysate samples were taken during IS. Following the completion of IS, rats were placed back in microdialysis bowls and four post-stress samples were collected from each rat. The perfusion flow rate was lowered to 0.2 μl/min until further testing 24 h later. Dialysates were immediately placed in a −80°C freezer until analysis. Samples were analyzed with high-pressure liquid chromatography using standard methods.

#### Juvenile social investigation with BLA 5-HT microdialysis

Twenty-four hours after IS, rats underwent a final JSI test with concurrent BLA 5-HT microdialysis. Green laser light was not delivered during this part of the experiment. Two hours prior to sample collection, The flow rate of the Ringer’s solution was increased to 1.5 μl/min. Samples were collected every 20 min. Four baseline 5-HT samples were taken prior to the JSI test. Eight minutes into the fifth sample, a juvenile was added to the microdialysis bowl for a total of 5 min. JSI testing was performed as described above. The juvenile was removed following the 5 min test and placed back in its home cage. Three samples were taken following juvenile social investigation. Dialysates were immediately placed in a −80°C freezer until analysis with high-pressure liquid chromatography (Christianson et al., 2010).

#### 5-HT quantification

5-HT concentrations were determined, as previously described (Amat et al., 1998), by HPLC with electrochemical detection. The system consisted of an ESA 5600A Coularray detector with an ESA 5014B analytical cell and an ESA 5020 guard cell. The column used was an ESA MD-150 (C-18; 3 μm; 150 × 3.2 mm), which was maintained at 40°C, and the mobile phase was the ESA buffer MD-TM. The analytical cell potentials were kept at −100 and +200 mV, and the guard cell was kept at +220 mV. Dialysate (25 μl) was injected with an ESA 542 Autosampler that kept the dialysates at 6°C. The 5-HT detection limit was 30 fg. External standards (Sigma-Aldrich) were run each day to quantify 5-HT by means of peak height and using ESA software.

### Experiment 3: effect of stress on Fos activation in the lateral habenula

#### Stressor controllability

In order to determine whether the LHb is differentially activated by controllable versus uncontrollable stressors, rats were exposed to either ES or IS in Plexiglas boxes (14 × 11 × 17 cm) with a wheel mounted on the front wall, as previously described (Baratta et al., 2009). Rats were run in yoked pairs (ES and IS) in 100 trial sessions (×2 h). Shock was delivered to the tail of the rat with increasing intensity as the shock session progressed (33 trials at 1.0 mA, 33 trials at 1.3 mA, and 34 trials at 1.6 mA). The average time between shocks was 90 s. Tailshock was terminated for both rats (ES and IS) when the ES rat achieved a specific wheel-turn requirement (Baratta et al., 2009). Therefore, onset, offset, and intensity were identical for both rats in each yoked pair. The initial wheel turn requirement was one-quarter of a complete revolution in <5 s. The response requirement increased with four successive successful wheel turns at each required fixed ratio interval. If the escape requirement was not reached within 30 s, the shock automatically terminated, and the escape requirement reverted to the previous, lower response requirement. Rats were removed from Plexiglas boxes and moved to their home cage immediately after the last tailshock.

#### Tissue preparation

Rats were deeply anesthetized with sodium pentobarbital (65 mg/kg) at either 0, 2, or 4 h following the last tailshock. HC rats that received no tailshocks were killed at the same time as experimental rats. Rats were transcardially perfused with 100 ml of ice-cold 0.9% saline solution, immediately followed by 250 ml of 4% paraformaldehyde in 0.1 m phosphate buffer (PB), pH ∼7.4. Brains were postfixed overnight in the same fixative and transferred to a 30% sucrose solution in 0.1 m PB, then were stored at 4°C until sectioning. Coronal brain sections containing MHb, the medial division of LHb (LHbM), and the lateral division of LHb (LHbL) were obtained at 40 μm. LHb tissue used for immunohistochemistry (IHC) was placed directly into a 24 well plate.

#### Immunohistochemistry

Staining for Fos was conducted using the avidin–biotin–horseradish peroxidase (ABC) method. Following a series of washes in 0.1 m PBS, sections were incubated in a 0.9% hydrogen peroxide solution in order to quench endogenous peroxidases. Then, sections were incubated for 24 h at room temperature (RT) with Fos primary antibody (1:15,000; Santa Cruz Biotechnology) in a blocking solution containing 2% normal goat serum (NGS), 0.5% Triton X-100 and 0.1% sodium azide. Following the primary antibody incubation, sections were incubated for 2 h at RT in biotinylated goat anti-rabbit secondary antibody (1:200; Jackson ImmunoResearch) in blocking solution. After a series of PBS washes, slices were then incubated in ABC for 1 h at RT. Next, sections were washed in 0.1 m PB, and then were exposed to a solution containing 3,3-diaminobenzidine, cobalt chloride, nickel ammonium sulfate, ammonium chloride, and glucose oxidase in PB. The peroxidase reaction was initiated by the addition of a glucose solution that reacted with the tissue for ∼7–10 min. The reaction was terminated by washing sections with PBS. Tissue was floated onto slide glass and coverslipped for later analysis.

#### Image analysis

Brain sections were observed using a bright-field microscope (Olympus BX-61, Olympus America) and analyzed using Olympus Suite Software (Olympus America). All digital images were captured using a 10× objective. Separate images of MHb, LHbM, and LHbL (AP −3.2 to −3.4 mm relative to bregma) were taken using parameters similar to those previously published (Brown and Shepard, 2013; Sego et al., 2014; Paxinos and Watson, 2007). Fos-stained nuclei in each subregion of the habenula were observed as dark brown or black round/ovoid spots. For each subject, two separate counts were taken from different slices within the section of habenula tissue spanning −3.2 to −3.4 mm AP relative to bregma. The two counts for each subregion were averaged and used for statistical analysis.

### Experiment 4: effect of stress on Fos activation in dorsal raphe-projecting lateral habenula neurons

#### Microinjection of retrograde tracer

Animals were anesthetized with isoflurane (Webster Veterinary). A small circular window (1 × 1 mm) was drilled to allow for the penetration of a needle (31 gauge, 45° tip angle) attached to a 10 μl Hamilton syringe. Using a stereotaxic instrument, the needle was directed to the DRN (AP, −8.0 mm from bregma; DV, −6.7 mm from skull surface; ML, 0.0 mm relative to midline). Two hundred nanoliters of a 2% solution of Fluoro-Gold (FG; Fluorochrome) dissolved in a 0.9% saline buffer was injected over the course of 3 min and allowed to diffuse for an additional 10 min using a microinjection pump (UMP3-1, World Precision Instruments). The scalp incision was closed using Vetbond (3M). Two weeks after FG microinjection, rats received ES, IS, or HC. FG microinjections were considered successful if the expression was visibly confined to DRN in coronal sections of brain obtained after completion of the experiment.

#### Stressor controllability

Rats were exposed to the same stressor controllability procedure described above in the methods of Experiment 3.

#### Tissue preparation

Coronal brain sections were taken at 40 μm, and habenula tissue used for IHC was placed directly into a 24 well plate. DRN tissue was mounted onto glass slides and coverslipped with VectaShield (Vector Laboratories) mounting medium. DRN tissue sections were later examined for confirmation of FG injection.

#### Immunohistochemistry

Staining for Fos was performed using a general immunofluorescence protocol. Following a series of washes in 0.01 m PBS containing 0.5% Triton X-100, slices were incubated overnight in a PBS blocking solution containing 2% NGS, 0.5% Triton X-100, and 2.5% bovine serum albumin at 4°C. Then, slices were washed in PBS and incubated for 24 h at RT in rabbit polyclonal primary antibody (1:2000; Santa Cruz Biotechnology) in blocking solution. After a series of PBS washes, slices were incubated for 2 h at RT in Alexa Fluor 546 goat anti-rabbit secondary antibody (1:250; Life Technologies). After a series of PBS washes, tissue was floated onto slide glass and coverslipped.

#### Image analysis

Brain sections were observed using an epifluorescence microscope (Axio Imager Z1, Zeiss), and images were captured using AxioVision software (Zeiss). All monochromatic digital images were captured using a 20× objective. For imaging and quantification of MHb, LHbM, and LHbL (taken between −3.2 and −3.4 mm AP relative to bregma), boundaries were based on those previously described (Brown and Shepard, 2013; Sego et al., 2014) in consultation with a brain atlas (Paxinos and Watson, 2007). FG-positive cell bodies in each subregion of the habenula were observed using the BFP filter set and were pseudocolored blue. Fos-stained nuclei were observed using a Cy3 filter set and were pseudocolored red. FG-positive and Fos-positive cells in each subregion were quantified and recorded separately. Colocalization of FG and Fos was reported when a magenta cell body representing the intermixture of the two fluorophores was observed and verified to be overlapping FG and Fos. For each subject, two separate counts were taken from different sections within the section of habenula tissue spanning −3.2 to −3.4 mm AP relative to bregma. The two counts for each subregion were averaged and used for statistical analysis.

### Statistical analysis

In Experiment 1, statistical analysis of JSI was performed using one-way ANOVA. Electrophysiological data were analyzed using one-way repeated-measures ANOVA. Both analyses were followed by Tukey’s *post hoc* analysis method. In Experiment 2, analysis of BLA 5-HT was performed using one-way repeated-measures ANOVA followed by Sidak’s *post hoc* method. The area under the curve (AUC) was computed relative to BLA 5-HT values, and statistical analysis was performed with an independent-samples *t* test. Analysis of JSI behavioral data was performed using independent-samples *t* test. Linear regression was used to correlate BLA 5-HT levels with JSI behavioral data. In Experiments 3 and 4, analysis of Fos (Experiments 3 and 4) and FG (Experiment 4) expression in local subregions of the habenula and treatment (ES, IS, and HC) was performed using two-way ANOVA followed by Tukey’s HSD *post hoc* test. All statistical analyses and graphing were performed using Prism software (GraphPad). The significance level was established at *p* < 0.05.

## Results

### Experiment 1: optogenetic inhibition of lateral habenula prevents the anxiety-like behavioral state that typically follows inescapable shock

*In vivo* single-unit recordings performed under urethane anesthesia (*n* = 2 subjects) demonstrated reliable silencing of LHb spontaneous spiking activity in response to 10 s of continuous green light delivery (Fig. 1*A*, top left). We successfully recorded from 36 neurons (rat 1, *n* = 14; rat 2, *n* = 22) in the LHb that met our criteria for inclusion (i.e., histological verification within LHb, expression of EYFP reporter in LHb in recorded region, firing rate >1 Hz) displaying an average firing rate of 7.9 ± 1.4 Hz. Using a one-way ANOVA to assess firing in the baseline, laser presentation and postlaser recovery periods followed by appropriate *post hoc* tests, we found that 86.1% (31 of 36 cells) showed a significant decrease in activity in response to laser illumination, while very few showed either no change in activity [8.3% (3 of 36 cells)] or an excitatory response [5.6% (2 of 36 cells); Fig. 1*A*, top right]. A perievent raster plot and histogram demonstrate decreased neural activity in a representative neuron in response to continuous laser illumination (Fig. 1*A*, middle).

We next determined whether these cells showed either rapid (<2 s) or slower (>2 s) latency in altered neural responding caused by the light presentations. A majority of neurons [61.1% (22 of 36 neurons)] showed rapid-onset changes in firing, although this population of rapid changes was exclusively inhibitory (22 of 22 neurons), with no cells showing rapid excitatory responses (0 of 22 neurons). The average latency to inhibition for the rapid-onset neurons was 0.75 ± 0.13 s, and it identified a second population of slower-onset neurons [30.6% (11 of 36 total cells)] that was comprised of both inhibitory [81.8% (9 of 11 slow-onset cells)] and excitatory [18.2% (2 of 11 slow-onset cells)] populations. These cells showed baseline firing rates that were similar to those of the rapid-onset populations (slow onset, 8.2 Hz; rapid onset, 7.9 Hz; unpaired *t*_(31)_ = 0.34, *p* = 0.73), although the average latency to significant deflection following laser onset was (by definition) significantly slower (slow onset, 2.5 s; rapid onset, 0.75 s; unpaired *t*_(31)_ = 8.26, *p* < 0.0001). However, within the slow-onset neurons, there was no difference in onset latency between inhibitory (2.4 s) and excitatory (2.8 s) responses (unpaired *t*_(9)_ = 1.24, *p* = 0.24). Given this distribution, we estimate that the rapid-onset neurons reflect the population of neurons that directly express NpHR [61.1% (22 of 36 neurons)], while the slower-latency onset neurons may reflect more secondary network effects [30.6% (11 of 31 neurons)] with the remaining neuron [8.3% (3 of 36 neurons)] unaffected directly or indirectly by light.

As a result, the net population activity of the LHb following laser presentation was strongly inhibitory. The *z*-normalized average firing rate data for all valid neurons (*n* = 36) displayed significantly decreased activity in the presence of the laser (*F*_(2,64)_ = 36.6, *p* < 0.00001, one-way ANOVA; Fig. 1*A*, bottom). *Post hoc* tests indicated that firing during the laser was significantly inhibited relative to both the prelaser baseline (*p* < 0.0002, Tukey’s *post hoc* test) and the postlaser recovery period (*p* < 0.0002, Tukey’s *post hoc* test), although there was no difference between the baseline and recovery periods (*p* = 0.77). Control recordings performed in a rat previously injected in the LHb with eYFP confirmed that light delivery alone had no effect on neural activity (*n* = 12 cells recorded; 0 of 12 cells showed significant change in firing rate related to green laser delivery).

In a separate cohort, we sought to determine the role of the LHb in IS-induced anxiety-like behavior measured during subsequent JSI (Fig. 1*B*). Functional NpHR expression was evident in the LHb 3–4 weeks after the initial viral injection (Fig. 1*B*, top). In Figure 1*B*, the top left shows bilateral NpHR expression (4×), and the top right shows NpHR expression and tissue damage from optical fiber implantation, denoted by an inverted arrow (10×). Twenty-four hours prior to IS or HC, rats underwent a baseline JSI test. Rats injected with eYFP or NpHR received IS (*n* = 10 and 11, respectively) with light delivery only during the tailshock portion of the IS session (∼8 min of total light delivery). HC rats previously injected with eYFP or NpHR (n = 10/group) received light delivery for an identical period of time/temporal pattern in their home cage. Twenty-four hours after IS or HC, rats underwent a final JSI test. Social investigation scores are reported as a percentage of the baseline social investigation (Fig. 1*B*, middle). NpHR-induced silencing of the LHb in HC (HC NpHR) had no effect on JSI. JSI was significantly reduced in rats previously injected with eYFP that received IS (IS eYFP). Remarkably, optogenetic silencing of the LHb during IS (IS NpHR) prevented reduced JSI. A two-way ANOVA revealed a main effect of stress treatment (*F*_(1,37)_ = 34.56, *p* < 0.0001), virus type (*F*_(1,37)_ = 9.193, *p* < 0.01), and a stress treatment × virus type interaction (*F*_(1,37)_ = 20.00, *p* < 0.0001). Tukey’s *post hoc* analysis revealed that IS reduced social interaction compared to HC (*p* < 0.0001). Tukey’s *post hoc* analysis also revealed that optical silencing (NpHR) of the LHb during HC did not affect social interaction (*p* = 0.56); however, silencing during IS restored social interaction to HC levels (*p* < 0.0001). These data indicate that LHb activity during IS is necessary for IS to increase anxiety-like behavior during JSI. Only rats with accurate optical fiber placement and bilateral virus expression in the LHb were included in the results (Fig. 1*B*, bottom).

### Experiment 2: optogenetic inhibition of lateral habenula during inescapable tailshock prevents the typical increase in BLA 5-HT observed during inescapable tailshock and subsequent juvenile social investigation

To determine the role of the LHb in the 5-HT alterations produced by IS, BLA 5-HT microdialysis samples were collected during IS and JSI from rats that received NpHR silencing or eYFP light delivery during IS. The BLA was chosen for the site of microdialysis based on previous work from Christianson et al. (2010) demonstrating that exaggerated 5-HT release and activation of BLA 5-HT 2C receptors mediate the anxiety-like behavior observed 24 h after IS during JSI. Rats previously injected with NpHR or eYFP in the LHb were implanted with bilateral optical fibers in the LHb and a unilateral microdialysis probe in the BLA (Fig. 2*A*). Figure 2*B* (left) shows extracellular levels of 5-HT as a percentage of baseline during IS in animals that received either LHb silencing with NpHR or eYFP light delivery during IS (*n* = 8 and 7, respectively). Also depicted is the AUC relative to baseline samples for the two groups (Fig 2*B*, right). Figure 2*C* (left) shows extracellular levels of 5-HT during JSI for animals that previously received either NpHR silencing of LHb or light delivery during IS. Also shown is the area under the curve relative to baseline samples for the two groups (Fig 2*C*, right). IS produced a robust increase in BLA 5-HT in rats injected with eYFP, which persisted after IS ended and the animals were returned to the microdialysis bowls. NpHR silencing of the LHb during IS almost completely prevented the increase in BLA 5-HT produced by IS. One-way repeated-measures ANOVA identified a main effect of time (*F*_(12,144)_ = 5.970, *p* < 0.0001), virus type (*F*_(1,12)_ = 20.29, *p* < 0.001), and a time × virus type interaction (*F*_(12,144)_ = 3.360, *p* < 0.001). *Post hoc* analysis revealed that NpHR silencing of LHb during IS prevented the typical increase in BLA 5-HT during IS at stress time points S1 (*p* < 0.0001), S3 (*p* < 0.05), S4 (*p* < 0.05), and S5 (*p* < 0.05), and following stress at post-stress time points P2 (*p* < 0.01), P3 (*p* < 0.05), and P4 (*p* < 0.05). An unpaired *t* test revealed that the area under the curve for IS rats that received NpHR silencing of LHb was significantly less (*p* < 0.01) than that for eYFP rats, which indicates lower relative BLA 5-HT levels in rats that received LHb silencing during IS. Basal BLA 5-HT levels did not differ between groups prior to JSI [mean (SEM): IS plus eYFP, 0.144 (0.033); IS plus NpHR, 0.118 pg/20 μl (0.019 pg/20 μl; *p* = 0.503, unpaired *t* test). Figure 2*B* shows BLA 5-HT levels during JSI. JSI produced a robust increase in BLA 5-HT levels in rats injected with eYFP that had previously received IS. NpHR silencing of the LHb during IS prevented this increase in IS-induced BLA 5-HT levels during later JSI. One-way repeated-measures ANOVA identified a main effect of time (*F*_(7,91)_ = 8.361, *p* < 0.0001), virus type (*F*_(1,13)_ = 13.39, *p* < 0.01), and a time × virus type interaction (*F*_(7,91)_ = 7.209, *p* < 0.0001). *Post hoc* analysis revealed that NpHR silencing of LHb during IS prevented the typical increase in BLA 5-HT levels during later JSI (*p* < 0.0001). An unpaired *t* test revealed that the area under the curve during JSI for IS rats that received NpHR silencing of LHb was significantly lower (*p* < 0.05) than that for eYFP rats. Consistent with results described in Experiment 1, an unpaired *t* test showed that rats that received NpHR silencing of the LHb during IS display significantly higher social investigation scores than rats who received light delivery only during IS (*p* < 0.001; Fig. 2*D*, left). BLA 5-HT levels measured during JSI were negatively correlated with social investigation scores (Fig. 2*D*, middle), such that higher BLA 5-HT levels resulted in lower JSI (*r* = −0.6257, *p* < 0.05). Only animals with accurate BLA microdialysis probe placement were included in statistical analysis (Fig. 2*D*, right).

### Experiment 3: escapable and inescapable shock equally increase Fos expression in the medial division of the lateral habenula

**Figure 3.**
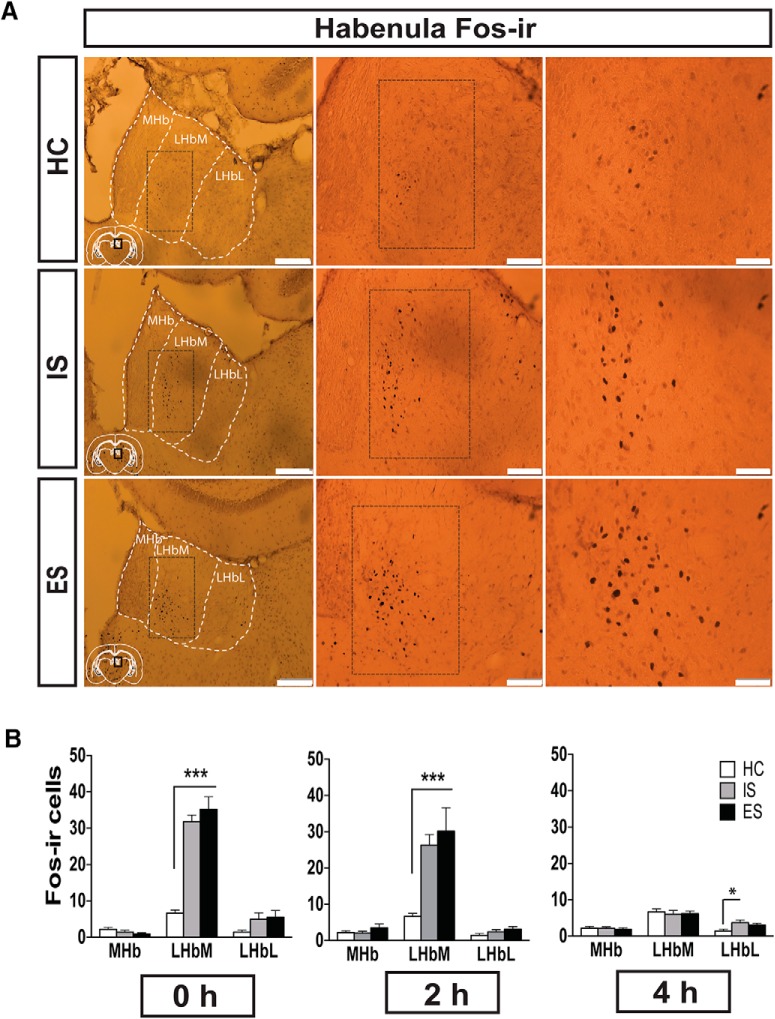
Stress, per se, increases Fos-IR in the LHbM. ***A***, Photomicrographs of Fos-IR in the habenula from rats that were killed 2 h after receiving HC (top), IS (middle), or ES (bottom). Images are shown at 10× (left), 20× (middle), and 40× (right) magnification. Scale bars: left, 200 μm; middle, 100 μm; and right, 50 μm. ***B***, Fos-IR was examined in the habenula at 0, 2, and 4 h following HC, IS, or ES (left to right). *n* = 8/group, two habenula sections/rat at all time points.

To determine whether LHb activity during tailshock is modulated by the controllability of the stressor, Fos protein immunoreactivity (Fos-IR) was examined in the MHb, LHbM, and LHbL in rats randomly assigned to HC, IS, or ES (Fig. 1). Rats were killed at 0, 2, and 4 h following the last tailshock (*n* = 8/group). A single home cage control group was used for all time points. HC rats remained in their cage but were placed in the same room in which stress occurred for ES and IS rats for ∼1.5 h (length of a typical stress session) and were killed 2 h following the end of this period of time. Representative images show that IS and ES, relative to HC, increased Fos-IR in the LHbM at 2 h post-stress (Fig. 3*A*). Data for the time course of stress-induced Fos-IR is shown in Figure 3*B*. ES, IS, and HC rats all showed very little Fos-IR in the MHb, a site previously shown not to be involved in the stress response, at 0, 2, or 4 h after tailshock. Similarly, very little Fos-IR was observed in the LHbL; however, one-way ANOVA revealed a significant effect of group at 4 h post-stress (*F*_(2,21)_ = 3.957, *p* < 0.05). Tukey’s *post hoc* analysis revealed that IS increased LHbL Fos-IR compared with HC (*p* < 0.05). Fos-IR in the LHbM at 0, 2, and 4 h after the last tailshock was analyzed using one-way ANOVA, which revealed a significant effect of group at 0 h (*F*_(2,21)_ = 37.94, *p* < 0.0001). *Post hoc* analysis revealed that the ES and IS groups showed enhanced total Fos-IR in the LHbM at 0 h compared with HC (*p* values < 0.0001); however, the two stress groups did not differ from one another. Similarly, the mean number of Fos-IR cells 2 h following the last tailshock was analyzed using one-way ANOVA, which revealed a significant effect of group (*F*_(2,21)_ = 9.34, *p* < 0.01). ES and IS groups showed enhanced total Fos-IR in the LHbM compared with HC (*p* value < 0.01); however, the two groups did not differ from one another (*p* = 0.52). By 4 h following the last tailshock, ES and IS Fos levels returned to HC level. These data suggest that stress, per se, activates the LHbM and LHbL, but that the structure does not appear to be differentially regulated by controllability of the stressor.

### Experiment 4: escapable and inescapable shock increase Fos expression in dorsal raphe-projecting lateral habenula neurons

Fos-IR and FG were examined in IS, ES, and HC animals perfused 2 h following the last tailshock (*n* = 12/group). This time point was chosen based on previous experiments (Baratta et al., 2009), as well as the initial time course study performed in Experiment 3, which revealed increased Fos-IR in the IS and ES groups relative to HC at that time point. There was not a significant difference in total FG-IR within the habenula between treatment groups. Total Fos-IR was examined in each subregion of the habenula using a one-way ANOVA, which revealed a significant effect of group on Fos-IR in the LHbM (*F*_(2,33)_ = 25.30 *p* < 0.0001) and LHbL (*F*_(2,33)_ = 5.193, *p* < 0.05). *Post hoc* analysis revealed that IS and ES enhanced Fos-IR in the LHbM, relative to HC (*p* values < 0.0001). *Post hoc* analysis also revealed that ES enhanced Fos-IR in the LHbL, relative to HC (*p* < 0.01). Total Fos-IR did not differ between ES and IS in either the LHbM or LHbL. Next, the percentage of DRN-projecting LHb neurons was examined using one-way ANOVA, which revealed a significant effect of group on Fos-IR in DRN-projecting LHbM cells (*F*_(2,33)_ = 14.89, *p* < 0.0001). *Post hoc* analysis revealed that ES enhanced Fos-IR in DRN-projecting LHbM cells compared with HC (*p* < 0.0001). Similarly, IS enhanced Fos-IR in DRN-projecting LHbM cells compared with HC (*p* < 0.001). These data suggest that stress per se activates the LHb–DRN pathway, and that the pathway is not differentially activated by controllable versus uncontrollable stress.

## Discussion

**Figure 4.**
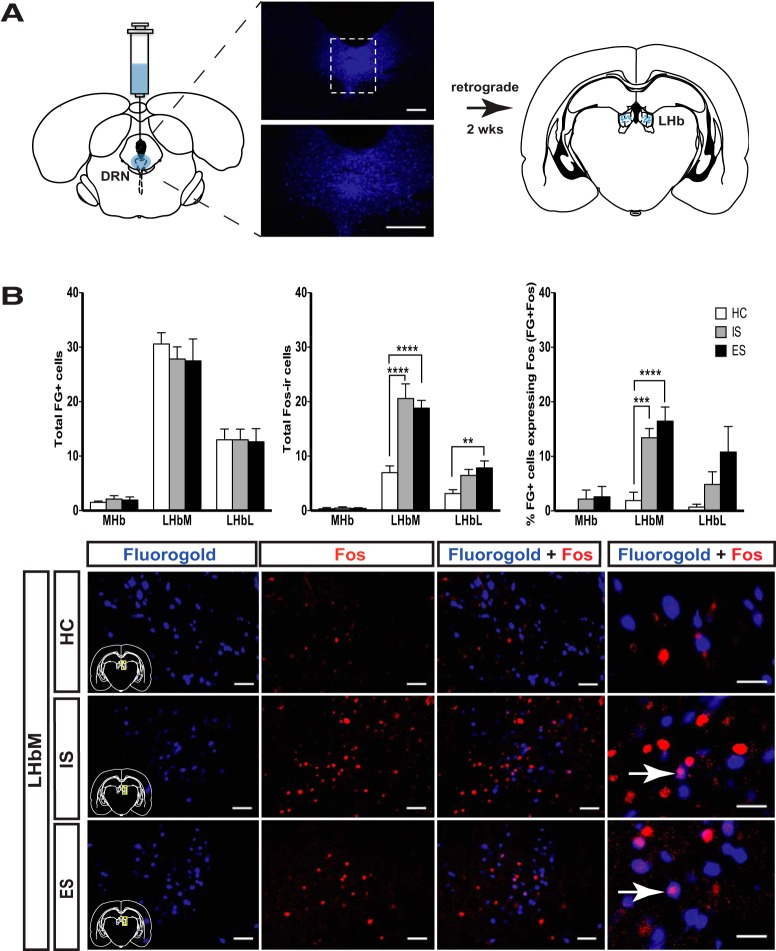
Stress, per se, increases Fos-IR in the LHb–DRN pathway. ***A***, Schematic showing FG injected directly into the DRN prior HC, IS, or ES (left). Photomicrographs show the site of injection at 4× (top) and 10× (bottom) magnification. Scale bars, 200 μm. Fluoro-Gold traveled retrogradely to the LHb prior to rats receiving HC, IS, or ES (right). ***B***, Two weeks after Fluoro-Gold injection, rats received HC, IS, or ES and were killed 2 h later. Total FG (left), Fos-IR (middle), and the percentage of FG cells also expressing Fos (FG+Fos; right) were quantified in the MHb, LHbM, and LHbL (top). The total number of FG-labeled cells in each habenula subregion (FG+Fos) did not differ across treatment groups (left). Total Fos-IR was increased in the LHbM and LHbL of rats that received either IS or ES (middle). Similarly, activation of the LHb–DRN pathway (% FG+Fos) was increased in rats that received either IS or ES (right). Photomicrographs (bottom) of representative LHbM tissue samples are shown for rats that received HC (top), IS (middle), or ES (bottom). Images in the first three panels are taken at 20× (scale bar, 50 um) and magnified to 40× at the far right (scale bar, 50 μm). FG reliably labeled cell bodies in the LHbM 2 weeks after injection (left). Increased Fos-IR is evident in rats that received IS or ES, relative to HC (middle). IS and ES also increased LHb–DRN pathway activation relative to HC, as measured by the colocalization of FG and Fos-IR (right). Arrows indicate colocalized FG and Fos-IR (magenta spot). *N* = 12/group, two habenula sections/rat.

Here we provide clear evidence that the behavioral and neurochemical consequences of DRN activation during IS requires LHb activation, a structure known for its role in regulating monoaminergic brainstem structures (Shabel et al., 2012). Optogenetic silencing of the LHb restricted to the tailshock intervals of an IS session blocked the typical increase in anxiety-like behavior observed 24 h later in the JSI test. Furthermore, this same manipulation prevented IS-induced BLA 5-HT release during both IS and subsequent JSI. Since the presence versus the absence of behavioral control during tailshocks modulates the behavioral and DRN-activating impact of the stressor, it was important to determine whether both IS and ES activate the LHb and LHb–DRN pathway, and if so whether they do so to the same degree. Both IS and ES resulted in a similar increase in Fos protein in the LHb. It should be noted that there was an increase in Fos protein in the LHbL 4 h after the final tailshock in rats that received IS, relative to HC; however, there was no difference in Fos protein in the LHbL between rats that received IS versus those that received ES. Similarly, a modest, albeit significant, increase in Fos protein levels was also observed in the LHbL of rats that received ES (Fig. 4*B*, middle). Interpreting how this difference influences the LHb–DRN circuit is difficult without knowing whether these neurons project to the DRN. The subtle differences observed between the two experiments analyzing Fos protein within the LHbL might be due to the different sample sizes used in the two experiments. IS and ES resulted in an equivalent increase in Fos protein levels, specifically in LHbM neurons that project to the DRN. A trend toward increased Fos protein levels in LHbL neurons that project to the DRN was observed in rats that received IS and ES. It should be noted that the total number of FG-labeled cells in the LHbL is low, relative to the total number of FG-labeled cells in the LHbM, and so a small change in Fos protein within FG-labeled LHbL cells will result in a larger increase in the percentage of cells expressing both FG and Fos. The findings of the present study have strong implications regarding the neural circuitry underlying both the behavioral and neurochemical consequences of IS and help to parcel out neural circuit elements relevant to stress-related psychiatric disorders, such as depression and anxiety.

Our results are consistent with prior research (Wirtshafter et al., 1994; Amat et al., 2001; Brown and Shepard, 2013; Sachs et al., 2015), and are more detailed and definitive with regard to the importance of an LHb–DRN circuit in mediating behavioral responses to acute stress. Amat et al. (2001) found that electrical lesion of the habenular complex prior to IS prevented IS-induced increases in DRN 5-HT levels and poor escape performance in a shuttlebox task 24 h later. However, lesions in this study often included portions of the MHb, would have damaged fibers of passage, and were agnostic to cell type, and it was also not determined whether this same manipulation affects extracellular 5-HT levels during subsequent behavioral testing. In addition, Amat et al. (2001) did not examine LHb activation. The present study used optogenetic tools to target and reversibly silence LHb pyramidal neurons with high temporal specificity (Gradinaru et al., 2010), which enabled real-time manipulation of LHb activity only during the tailshock itself while performing simultaneous microdialysis in the BLA, a downstream circuit element that is critical for the expression of IS-induced anxiety-like behavior (Christianson et al., 2010). Thus, the LHb was inactivated for only 500 s of the ∼100 min IS treatment session. There are two main limitations of the current study that should be acknowledged. The first pertains to the lack of negative control groups in which light was delivered to the LHb while performing microdialysis in the absence of IS. However, it should be noted that the HC eYFP and HC NpHR groups displayed very similar behavior during JSI, and so it is unlikely that LHb silencing in the absence of IS would have a profound effect on 5-HT release within the BLA. The second limitation pertains to the site that was chosen for 5-HT sampling. In addition to the DRN, the median raphe also projects to the BLA, and so it may provide an additional source of 5-HT measured in the BLA (Azmitia and Segal, 1978). Consistent with other reports demonstrating increased Fos induction following stress per se, both IS and ES (two different types of stressors) yielded significant activation of the LHb (Chastrette et al., 1991; Nagao et al., 1993; Wirtshafter et al., 1994; Brown and Shepard, 2013). This was also observed in the LHb–DRN pathway. To our knowledge, this is the first study to measure Fos induction restricted to DRN-projecting LHb neurons following a stressor. While previous work has demonstrated that IS, relative to ES, produces greater activation and Fos protein levels in the DRN (Maswood et al., 1998; Grahn et al., 1999), measuring Fos protein levels in the DRN following optogenetic manipulation of the LHb would provide additional information regarding whether LHb silencing in itself is sufficient to prevent IS-induced DRN activation.

The present data have important implications for a long-standing issue concerning the mechanisms that mediate the modulatory effects of behavioral control. As often demonstrated, IS activates and sensitizes DRN 5-HT neurons, and leads to a variety of anxiety-like and depression-like behavioral changes that are caused by these DRN alterations (for review, see Maier and Watkins, 2005). However, exactly equal ES neither activates the DRN nor produces these behavioral changes. There are two possibilities with regard to the differential DRN 5-HT activation produced by ES and yoked IS, as follows: (1) IS could provide greater excitatory input to the DRN than does ES; or (2) IS and ES could each provide excitatory input, but ES also leads to inhibitory input that IS does not. Of course, both could be true to some degree. A variety of evidence (for review, see Maier, 2015) suggests that ES, but not IS, does, in fact, lead to inhibitory input initiated in the medial prefrontal cortex. However, whether IS and ES might still produce differential excitatory input has not been resolved. One strategy is to examine structures that provide major excitatory input to the DRN. Here we examined the LHb because it provides a strong glutamatergic input to the DRN (Lee et al., 2003; Aizawa et al., 2012; Sego et al., 2014) and found that optogenetic silencing of the LHb during IS did indeed block the 5-HT increase produced by IS as well as the behavioral effect of IS measured here. These data further support the role of 5-HT in mediating the behavioral effects of stress, but more importantly, ES led to as large an Fos increase in LHb neurons that specifically project to the DRN as did IS. These data are the strongest to date in suggesting that ES and IS provide equivalent excitatory input to the DRN. That is, the pattern suggests that stressors per se activate the DRN, without regard to psychological factors, such as controllability, and that the presence of control then actively inhibits the DRN.

A long-standing disagreement as to whether LHb inputs to the DRN are excitatory or inhibitory requires comment. Some studies have suggested that electrical stimulation of the LHb in anesthetized rodents results in a transient postsynaptic inhibition of putative DRN 5-HT neurons (Aghajanian and Wang, 1977; Park et al., 1987; Varga et al., 2003). Others have indicated that electrical stimulation of the LHb activates DRN 5-HT neurons (Ferraro et al., 1996) and results in increased extracellular 5-HT in the striatum of anesthetized rats (Kalén et al., 1989). There are numerous procedural differences between these studies, and the present data do not resolve the issue. The results of the current study, however, suggest an excitatory role for the LHb in driving activation of the DRN, as silencing the LHb during IS prevented both the behavioral and neurochemical consequences of the stressor, which typically depend on DRN activation. The present study is the first to assess the effects of reversible inactivation of the LHb during uncontrollable stress on 5-HT release in a DRN projection region during the stressor and also during a subsequent behavioral test.

There is also the possibility that the modulation of DRN activity during stress is due to an indirect projection beginning in the LHb, rather than by a direct projection to the DRN. Indeed, the LHb projects to the rostromedial tegmental nucleus (RMTg), which in turn projects to the DRN (Lammel et al., 2012; Sego et al., 2014). However, RMTg projections to the DRN are enriched in GAD67 mRNA and synapse in a region histochemically characterized as 5-HT poor but glutamate enriched (Sego et al., 2014). Direct projections from the LHbM to the DRN synapse instead in a region enriched in 5-HT (Sego et al., 2014). While there is a possibility that the RMTg is a proximal mediator of the DRN response to stress, this is unlikely. In the present study, IS increased extracellular 5-HT, and LHb silencing prevented this increase. Moreover, enhanced Fos expression following IS and ES was almost exclusively confined to the LHbM, with only a modest increase observed in the LHbL. Consistent with this observation are data from previous anatomical tracing studies indicating that the LHbM sends strong projections primarily to the DRN, while the LHbL sends the majority of its projections to the RMTg (Sego et al., 2014). Also, IS and ES increased Fos expression primarily in the LHbM and LHbM–DRN pathway, and so a critical role for the direct LHb–DRN pathway is suggested.

The vast majority of research involving the LHb has focused on its role in regulating midbrain dopamine (DA) release (for review, see Lecca et al., 2014; Proulx et al, 2014). Much of this work focuses on the input from the LHb to the primarily GABAergic RMTg, which inhibits ventral tegmental area DA release during negative reward prediction error and punishment (Ji and Shepard, 2007; Jhou et al., 2009; Matsumoto and Hikosaka, 2009; Lammel et al., 2012; Stopper et al., 2014). Because dysregulation of DA has been implicated in psychiatric disorders, including addiction, schizophrenia, and depression (for review, see Hikosaka, 2010), the LHb has gained popularity as a potential therapeutic target for drug addiction and depression-like behavior (Friedman et al., 2010; Sartorius et al., 2010; Christensen et al., 2013; Yadid et al., 2013). Surprisingly, very few studies have focused on the contribution of the LHb–DRN pathway to stress and stress-related psychiatric disorders, including anxiety and depression. The present findings indicate an important role for this pathway in the behavioral outcomes of stress and highlight the importance of increased effort in this area of research.

## Synthesis

Reviewing Editor: Daniela Schiller, Mount Sinai School of Medicine

Decisions are customarily a result of the Reviewing Editor and the peer reviewers coming together and discussing their recommendations until a consensus is reached. When revisions are invited, a fact-based synthesis statement explaining their decision and outlining what is needed to prepare a revision will be listed below. The following reviewers agreed to reveal their identity: Victor Viau, Klaus Miczek

The data shows that the lateral habenula (LHb) plays an important role during inescapable shock (IS) exposure to alter behavioral and 5-HT responses during a subsequent test of juvenile social interaction, but that the Fos activation of a LHb cells identified as projecting to the dorsal raphe nucleus is not distinguished by behavioral control (i.e. between animals experiencing inescapable and escapable shock exposure).

The results are exciting, but it would seem that these two pieces of information are explored rather separately from each other in the Discussion. Thus, the authors should provide some additional perspectives to underscore where and how the LHb might be involved in the dimension of behavioral control. This is particularly important given the fact that this study's findings are largely negative (at least as far as behavioral control is concerned), which appears to get lost amidst the current manuscript's detailed descriptions of the importance of ES vs. IS and references to the importance of the LHb. Thus, a more explicit discussion of where the LHb fits would be appropriate and helpful to readers.

Based on the Fos based data, restricted only to the LHb, the authors conclude these findings to reflect excitatory input to the DRN. Based on their limited survey, and without any measure of Fos induction in the DRN, the authors should also consider providing some caveats to this limited approach.

Figure 2 lacks a negative control entirely, it would have been nice to see home cage controls in Figure 2 to demonstrate that the observed effects are stress specific. This limitation could be explicitly acknowledged in the discussion.

It is of very slight concern that the data in 3B (middle panel) and 4B (middle panel) did not replicate. In 3B, no significant effect of IS or ES was observed in the LHbL at 2 hours, but in figure 4B, this effect was significant. Granted, there were more animals per group in 4B compared to 3B, but it begs the question of whether additional animals had been included in 3B, significant effects in the LHbL might have been observed at all timepoints.

Also in Figure 3, it appears that the significance of the increase in Fos-ir in the LHbL at 4 hours that is referred to in the text is not noted with a star in the figure. Although not justified, some might argue that the fact that a significant increase in Fos was observed in response to IS but not ES argues against the major conclusion of the paper. It may be helpful to address this point more directly with an additional sentence.

Similarly, the data in Fig 4B (far right - %FG+ Fos+ cells in the LHbL), while not significant, do reflect a trend. While statistically speaking, the LHbL data in the far left panel (Total FG+) and the far right panel (%FG/Fos) are equivalent (no difference), they certainly do not appear identical, so a few words of discussion seem warranted.

Finally, there are several typos:

Line 261: an extra "to the"

Line 112: avoid laboratory short-hand "lab chow"

Line 179: "binned"?

Line366: "...verified to be..."

Line 371: "analysis"
